# Emotional eating, internet overuse, and alcohol intake among college students: a pilot study with virtual reality

**DOI:** 10.3389/fnut.2024.1400815

**Published:** 2024-06-18

**Authors:** Carlos Marchena-Giráldez, Myriam Carbonell-Colomer, Elena Bernabéu-Brotons

**Affiliations:** Faculty of Education and Psychology, Universidad Francisco de Vitoria (UFV), Madrid, Spain

**Keywords:** emotional eating, alcohol intake, internet addiction, executive functions, virtual reality, impulsivity, depression

## Abstract

**Introduction:**

The term emotional eating (EE) describes the tendency to eat as an automatic response to negative emotions and has been linked to anxiety and depression, common symptoms among the university population. The EE tendencies have also been associated with excessive internet use and an increase in alcohol intake among young university students.

**Methods:**

The aim of this study is to examine the relationship between the tendency towards EE and other health-compromising behaviors, such as excessive internet use or high alcohol intake. Additionally, it aims to investigate the association of these risky behaviors with the participants’ performance level in a virtual reality (VR) task that assesses their executive functioning, and to assess impulsivity and levels of anxiety and depression.

**Results:**

The results associate EE with excessive internet (r = 0.332; *p* < 0.01). use but not with alcohol consumption. Alcohol consumption was not associated with anxiety, depression, or impulsivity, but it was related to altered executive functions in the VR task: flexibility and working memory explained 24.5% of the variance. By contrast, EE and internet overuse were not related to executive function but were associated with impulsivity, depression, and anxiety. Impulsivity and depressive symptoms accounted for 45% of the variance in EE. Depression, trait anxiety and impulsivity explained 40.6% of the variance in internet overuse.

**Discussion:**

The results reveal distinct patterns of psychological and neuropsychological alterations associated with alcohol consumption compared to emotional eating (EE) and excessive internet use. These findings underscore significant differences in the contributing factors between addictions and other substance-free addictive behaviors. For a deeper understanding of the various contributing factors to EE in college students, further research is recommended.

## 1 Introduction

Eating behavior is a multifaceted phenomenon influenced not only by biological or genetic factors, but also by environmental and psychological factors (1). For more than 30 years, studies have consistently highlighted the significance of self-regulation and emotion in shaping the emergence of unhealthy eating habits (2, 3). Stress and negative emotions are frequently linked to increased food intake (4). One of the most dysfunctional eating habits is emotional eating. The term *emotional eating* (EE) describes the propensity to eat as an instinctual reaction to unpleasant feelings (5) and implies a disproportionate intake of food, regardless of whether or not the individual is actually hungry, but in a nonpathological way. It differs from *binge eating disorder* (BED), which was officially recognized in 2013 in the DSM-5 as a separate eating disorder characterized by recurrent episodes of binge eating accompanied by a feeling of loss of control over eating, along with marked distress regarding binge eating, but without the compensatory mechanisms observed in anorexia or bulimia nervosa such as self-induced vomiting or excessive laxative use (6).

EE has been linked to symptoms of anxiety and depression in the general (non-clinical) population (7, 8). Episodes of emotional eating (EE) are typically accompanied by high-calorie, sugary, and/or high-fat food consumption. This eating pattern has been described as one behavioral mechanism linking depression to the development of obesity and abdominal fat (9). Individuals who are obese or overweight frequently experience this eating behavior pattern (10). Emotional eating was also strongly associated with psychological distress (11) and feelings of guilt or shame (12, 13).

University students exhibit elevated anxiety levels compared to the broader population with concerns about academic performance being a primary contributor to this distress (14, 15), and they also grapple with issues such as gaining autonomy from parents, financial constraints, and social competition or isolation, among various other concerns (16). Stress, anxiety, and depression emerge as prevalent psychological challenges faced during university education globally. Studies consistently indicate that university students tend to experience lower mental well-being compared to the general population (17, 18). When they fail to adapt adequately, they may exhibit maladaptive coping behaviors. Recent studies indicate a broad spectrum of risky behaviors among college students, such as heavy alcohol consumption and unhealthy eating patterns (19, 20). A considerable level of EE (29%) has been also found among the university population (8). Lack of effective stress management strategies can lead to resorting to unsuitable or harmful coping mechanisms like increased alcohol intake or EE. In a recent study, the frequency, and patterns of EE over the past 28 days were assessed among 335 female university students. The findings revealed that 51.3% of these episodes were associated with emotional states, notably anxiety (21), and it has been suggested that the intake of alcohol might serve as a substitute coping mechanism for managing negative emotions, particularly anxiety, replacing EE. A study employing person-centered longitudinal analysis over three years found that undergraduates tend to resort to excessive drinking as a means of relieving stress and managing social anxiety (22).

The existence of eating disorders, such as anorexia, bulimia, and binge-eating disorder, has also been associated with problematic internet use among university populations (23), suggesting that the duration of students’ internet usage influences eating behavior disorders (24). At a subclinical level, problematic internet and smartphone abuse has been linked to problems with self-control over eating and a greater propensity to consume automatically in reaction to negative emotions or sentiments in teenagers and young adults, as shown by a study involving 209 adolescents as part of a school-based mental health promotion program (25), consumption pattern previously described and known as EE (26). Since both EE and excessive internet use are probable products of a lack of emotional regulation, there is undoubtedly a connection between the two (27). Additionally, a recent study conducted with 551 adolescents aged 15–17 suggests that social pressure and anxiety may act as mediating factors in the association between EE and internet addiction (IA), particularly social media addiction via smartphones (28).

College students experience a phase of transition into adulthood where they have attained physical maturity but might not have achieved similar levels of psycho-social development (29). The onset of the college experience often brings about substantial lifestyle shifts, notably in dietary habits characterized by skipping meals, insufficient nutrition, and frequent consumption of fast food. Moreover, during this stage, the brain is highly susceptible to external influences, particularly in the final phases of neuromaturation, which represent a critical period for neurodevelopment (30). In associative areas, especially the prefrontal cortex, there is a notable increase in white matter between the ages of 18 and 22, which correspond to the first years of university (31). Additionally, throughout the first half of the first year of university, there have been changes found in the volume of grey matter (32). These changes have been linked to the executive function’s end of maturation, which occurs between the end of adolescence and the beginning of adulthood (33). The prolonged developmental phase explains the specific vulnerability of executive functions compared to other cognitive processes influenced by external factors, risky behaviors, or addictions. Executive functions (EF) are higher-level cognitive mechanisms that regulate lower-level cognitive processes and also impact behavioral control (34), and have an important role in the development of emotional regulation strategies: a heightened level of executive functions is somewhat connected to the effective implementation of emotion regulation strategies (35), and deficiencies in executive functions (working memory, planning, flexibility) are widespread among adolescents experiencing challenges in emotional regulation (36). Various studies have found alterations in the executive functions of university students associated with alcohol consumption (20, 30), abusive internet use (37), and EE (38). Regarding this, a systematic review demonstrates that proper functioning of executive functions is associated with adequate and correct self-regulation of eating behavior (2), and a longitudinal study conducted with 169 teens with type 1 diabetes shows that high levels of disordered eating practices in teenagers are associated with difficulties in EF (39).

In the evaluation of EF, discrepancies sometimes emerge between the scores obtained in traditional pen-and-paper tests and the performance of tasks in daily life. The utilization of virtual reality (VR) technology increasingly influences the process of neuropsychological assessment. By facilitating dynamic interaction and replicating three-dimensional environments, VR provides participants with an immersive experience that closely simulates real-life settings (40). VR also allows for the reproduction of multitasking environments, necessary to increase the ecological validity and predictive value of the functional performance of the individuals being evaluated (41).

Another relevant factor to consider in the increasing prevalence of these unhealthy and risky abusive behaviors among college students is impulsivity. Impulsivity results from a lack of inhibitory control, and it is defined as the tendency to act swiftly in response to any stimulus, whether internal or external, without prior evaluation of all available information and regardless of the potential consequences of the action. Numerous studies corroborate the pivotal role impulsivity plays in substance addictions (42), alcohol consumption (43), and it also appears to be a relevant factor in internet addiction (37, 44). Impulsivity has been directly linked to overeating (45), food addiction (46), and BED (47), serving as a mediating factor between EE and overweight (48).

The objectives of this study include:

1. To assess the levels of EE in a sample of college students and their relationship with other abusive behaviors, such as excessive internet usage and alcohol consumption.
2. To verify the relationship between these behaviors, impulsivity and executive functioning assessed through a virtual reality (VR) task.
3. To evaluate the distinct roles that depression and anxiety may play in EE, excessive internet usage and alcohol consumption.

## 2 Method

### 2.1 Participants

The sample consisted of 56 undergraduate students (age > 18), of whom 30.4% were men and 69.6% were women. The age range was between 18 and 26 years. The mean age of participants was 20.62 (SD = 2.4). All the participants were university students from Francisco de Vitoria University in Madrid, Spain. The students were selected through non-probabilistic and accidental sampling. Table 1 shows more details about sample characteristics. Inclusion criteria were not having any psychiatric diagnosis, not being receiving psychological treatment at the time of the study and not consuming psychoactive substances. The study protocol and design were approved by the Ethics Committee of the University Francisco de Vitoria, and it fully complied with the Helsinki Declaration.

**Table 1:** Description of the sample.

Variable	Variables	*%*
Study level	Graduate	12.3%
	Undergraduate	87.7%
Emancipated	No	78.9%
	Yes	21.1%
Sentimental state	Single	50.9%
	In a relationship	49.1%
Current on diet	Yes	10.5%
	No	89.5%
Eating disorder diagnosis	Yes	0%
	No	100%

### 2.2 Variables and instruments

*Sociodemographic information.* Participants provided information about their age, gender, and educational level through a questionnaire created *ad hoc*.

*Emotional eating* was measured using the Emotional Eater Questionnaire (EEQ) (49). The EEQ consists of 10 items on a 4-category Likert scale ranging from 0 to 3. The test provides a global score to distinguish between non-emotional eaters (score between 0–5), slightly emotional eaters (score between 6–10), emotional eaters (score between 11–20), and highly emotional eater (score between 21–30). The temporal stability shows a medium to high correlation in the test–retest average (*r* = 0.702; *p* < 0.001), and the internal consistency of the subscales ranges from *α* = 0.61 to *α* = 0.77. In our study, the internal consistency was *α* = 0.86.

*Internet addiction* was assessed using the Internet Addiction Test (IAT). This instrument was originally created in 1998 (50). For our study we used the Spanish version validated by Puerta-Cortés et al. (51). The IAT consists of 20 items with a Likert-type response format with 5 levels. The cutting point for identifying internet overuse is 40. The diagnostic validity showed 81% sensibility and 82.6% of specificity. Internal consistence values obtained in the validation study was good (*α* = 0.82). In the present study, internal consistence was also good (*α* =0.88).

*Alcohol consumption* was measured using the Alcohol Use Disorders Identification Test (AUDIT) (52). This questionnaire was composed of 10 items with a five-point (0–4) Likert scale as a response format, except for the last two items, which have three answer options (0–2). Problematic alcohol consumption is considered from 8 points. AUDIT showed high sensibility and specificity (0.90 and 0.80 respectively). The validation of the test on a Spanish university population (53) found the internal consistency of the AUDIT test to be *α* = 0.75. In the present study, the internal consistency for the AUDIT was also good (*α* = 0.83).

*Depression* was measured with the Beck Depression Inventory (BDI), revised and updated in 1996 (54) and validated in a Spanish sample (55). The BDI consists of 21 items on a 4-category Likert scale (0–3). The test score allows for classification into minimal depression (0–13), mild depression (14–19), moderate depression (20–28) and severe depression (29–63). The internal consistence of the BDI in previous studies was *α* = 0.83. In the present study, the internal consistence was good (*α* = 0.90).

*Anxiety* was assessed by using the State–Trait Anxiety Inventory (STAI), adapted to the Spanish population (56). This questionnaire consists of a 40-items scale divided into two parts: the first 20 items measure anxiety as state; and the las 20 items measure anxiety as trait. Each of the 20 items in each subsection uses a 4-category Likert scale ranging from 0 to 3. A reliability study showed high internal consistency (*α* = 0.93). In the present study, the internal consistency was also good (*α* = 0.81).”

*Impulsivity* was measured using the Barratt Impulsiveness Scale, a questionnaire that was originally created in 1987 and adapted to the Spanish population in 2001 (57). The scale consists of 30 items with 4 response options, from 1 (always) to 4 (never). Reliability analysis showed good values of internal consistency (*α* =0 0.81). In our study the internal consistency was adequate (*α* = 0.70).

*Processing speed, planification, working memory and flexibility* were measured with the Ice Cream Test (ICT), created by Giunti Psycometrics®, within Nesplora® package through virtual reality. This test has shown validity and reliability to measure executive functions (40). The task allows to measure attention, working memory, planification, flexibility and processing speed at the same time (58).

The participants were immersed in a VR experience where they had to assume the role of an ice cream vendor, following the instructions given at the beginning of the task. All task instructions are delivered audibly: “*you’ll be working at the ice cream shop for a while. Customers come in groups of four, and you must serve them according to your boss’s instructions. Call your boss, and he’ll provide you with his priorities for serving customers. Click on the phone to give him a call*.” The boss’s instructions outlined the order of attending to customers based on various criteria and the preparation of ice creams, which differed for each customer. Participants were provided with a recipe book, which they could consult whenever they deemed it necessary.

The test consists of 20 sequences divided into two rounds, A and B. Prior to the start of the task, all participants underwent a series of practice trials to ensure that the instructions were properly understood and that the task was manageable for each subject. In both parts A and B, the test operates with the same structure, environment, and task. Midway through the test, the initially learned series of ice creams changes, and a new set of ice cream variants must be learned to perform correctly in the second half of the test. The task requires to attend to customer requests and orders, utilize working memory to remember orders and manage inventory, plan, and organize tasks efficiently to serve customers. In round 2, participants must adapt to changing customer demands, demonstrating flexibility, and process orders quickly to serve customers effectively, thus measuring processing speed. In this way, upon finishing the test, four indices can be obtained for each participant: planning (correct assignments, assignment time, cognitive load), working memory (correct services without consultation), flexibility (interference between instructions, switching) and processing speed (duration of task completion).

The immersive and interactive nature of this VR experience can enhance the engagement of the assessment and potentially offer a more accurate representation of an individual’s executive functioning abilities in a real-world context.

### 2.3 Procedure

The study was conducted in two steps: first, using the Qualtrics platform,^1^ where participants completed the questionnaires. The second step took place in person, individually, in a calm and quiet environment. In the second step, participants completed the VR task (ICT). Participation was voluntary and required prior informed consent. Participants did not receive any rewards. This study received approval from the University Francisco de Vitoria Ethics Committee and was conducted in accordance with the principles of the Declaration of Helsinki.

### 2.4 Data analysis

Firstly, we obtained descriptive statistics for all the measures. To analyze the association between psychological and neuropsychological variables and emotional eating, internet overuse, and alcohol consumption, we calculated Pearson correlation coefficients. Finally, we conducted a series of stepwise linear regression models, introducing the significantly associated variables along with age and gender as explanatory variables. The data obtained in the study was analyzed using the Statistical Package for the Social Sciences 25.0 (SPSS) for Windows.

## 3 Results

### 3.1 Descriptive analysis and linear correlation among the studied variables

Table 2 displays the descriptive analysis of each measure. Scores exhibit moderate variability based on the range values, typical of a subclinical sample.

**Table 2:** Descriptive analysis of participants’ values for evaluated variables.

Measures	Variables	*M* [Range]	*SD*
EEQ	Emotional eating	9.8 [2–29]	5.83
IAT	Internet overuse	30.71 [4–63]	13.06
AUDIT	Alcohol consumption	5.64 [0–21]	4.8
BDI	Depression	12.41 [1–36]	9.57
STAI	Anxiety state	24.93 [11–36]	4.88
	Anxiety trait	28.57 [17–38]	4.42
BIS	Impulsivity	23.14 [8–37]	6.95
ICT	Processing speed 1	5.24 [3.24–5.74]	1.09
	Processing speed 2	5.62 [3.53–5.94]	1.28
	Planification	45.53 [39–72]	7.24
	Working memory	27.03 [17–28]	1.87
	Flexibility	−2.71 [−7–0]	1.35

AUDIT, Alcohol Use Disorders Identification Test; BDI, Beck Depression Inventory; BIS, Barratt Impulsiveness Scale; EEQ, Emotional Eater Questionnaire; IAT, Internet Addiction Test; ICT, Ice Cream Test; STAI, State–Trait Anxiety Inventory.

The Pearson correlation coefficients between each of the measures are presented in Table 3. Considering the main study variables, EE and the propensity for internet addiction are related. Trait anxiety and depression, along with increased impulsivity are associated with EE. These same variables and additionally the processing speed (measure round 2) were positively associated to the internet overuse score. Concerning alcohol consumption, processing speed (measured in round 2) also exhibited a positive association with AUDIT scores, albeit in a different context; meanwhile, working memory and flexibility demonstrated an inverse correlation with this variable. However, alcohol intake is not linked to higher levels of anxiety and depression.

**Table 3:** Correlation analysis: Pearson correlation coefficient between the measures.

Measures	1	2	3	4	5	6	7	8	9	10	11
1. EEQ											
2. IAT	0.332*										
3. AUDIT	0.216	−0.002									
4. BDI	0.548**	0.466**	0.155								
5. STAI (trait)	0.370*	0.448**	0.186	0.275*							
6. STAI (state)	0.249	0.050	0.111	−0.053	0.095						
7. BIS	0.449**	0.376**	0.212	0.075	0.270*	0.338*					
8. ICT Processing speed 1	0.002	0.168	0.149	0.023	0.219	0.040	0.239				
9. ICT Processing speed 2	0.058	0.129*	0.318*	0.173	0.139	0.068	0.073	0.638**			
10. ICT Planification	0.183	0.225	−0.052	0.164	0.191	−0.005	0.050	0.344*	0.307*		
11. ICT Working memory	−0.084	−0.045	−0.410**	−0.017	−0.044	−0.055	−0.303	−0.635**	−0.453**	−0.092	
12. ICT Flexibility	−0.223	0.062	−0.296*	−0.171	0.036	−0.082	0.000	−0.154	−0.547**	−0.084	0.153

AUDIT, Alcohol Use Disorders Identification Test; BDI, Beck Depression Inventory; BIS, Barratt Impulsiveness Scale; EEQ, Emotional Eater Questionnaire; IAT, Internet Addiction Test; ICT, Ice Cream Test; STAI, State–Trait Anxiety Inventory. *p < 0.05; **p < 0.01.

### 3.2 Explanatory models of emotional eating, internet overuse, and alcohol consumption

Considering the psychological and neuropsychological variables associated with the main study variables, a series of stepwise regressions were conducted. In these regressions EE, internet overuse and alcohol consumption were introduced as dependent variables and the previous variables with significant correlations were introduced as independent variables. In the same way, gender and age were also included as independent variables in the explanatory models. Table 4 shows the explanatory model of emotional eating that was obtained.

**Table 4:** Regression analysis: explanatory model of emotional eating.

Model	Variables	*b*	*t*	R adjusted
1	Depression	0.519	5.021**	31%
2	Depression	0.519	5.021**	31%
	Impulsivity	0.402	3.895**	16%

*p > 0.05; **p > 0.001.

For EE, depression and impulsivity resulted as explanatory variables. The combined model, comprising both variables, accounted for 45% of the variance in EE (*F* = 22.293; *p* > 0.001). Trait anxiety, gender, and age did not emerge as significant explanatory variables. For this model, the assumptions of linear regression were met. The Durbin-Watson statistic for residual independence was 2.047, and the collinearity criteria displayed appropriate values: tolerance level > 0.1 (1.00) and FIV < 10 (1.004).

Regarding internet overuse scores, Table 5 shows the explanatory model obtained. Levels of depression, trait anxiety, and impulsivity were significant explanatory variables. The complete model explained 40.6% of the variance in internet overuse (*F* = 11.412; *p* > 0.001). However, processing speed (round 2), gender, and age did not result as significant explanatory variables of internet overuse. Conditions for linear regression were also met. The Durbin-Watson test value was 1.711, and values for no collinearity were appropriate: tolerance level > 0.1 (0.85–0.932) and FIV < 10 (1.00–1.176).

**Table 5:** Regression analysis: explanatory model of internet overuse.

Model	Variables	*b*	*t*	R adjusted
1	Depression	0.377	3.339**	22%
2	Depression	0.377	4.339*	22%
	Trait anxiety	0.292	2.469*	12%
3	Depression	0.377	4.339*	22%
	Trait anxiety	0.292	2.469*	12%
	Impulsivity	0.245	2.137*	5%

*p > 0.05; **p > 0.001.

Lastly, Table 6 shows results of explanatory model of alcohol consumption. We identified working memory and flexibility as significant explanatory variables. However, processing speed (round 2), gender, and age were not significant. The complete model explained 24.5% of the variance in alcohol consumption (*F* = 8.428; *p* = 0.001). Similarly, the requirements for linear regressions were satisfactorily met. The Durbin-Watson test value was 2.057, and for no collinearity, we obtained tolerance levels >0.1 (0.978–1.000) and FIV < 10 (1.000–1.023).

**Table 6:** Regression analysis: explanatory model of alcohol consumption.

Model	Variables	*b*	*t*	R adjusted
1	Working memory	−0.389	−3.189*	18%
2	Working memory	0.389	−3.189*	18%
	Flexibility	−0.257	−2.083*	6%

*p > 0.05; **p > 0.001.

## 4 Discussion

Our findings uncover specific patterns of psychological and neuropsychological changes linked to EE and excessive internet use when contrasted with alcohol consumption. These differences suggest that in university students, there are diverse underlying mechanisms and behavioral pathways, shedding light on the unique impacts of each behavior on cognition and mental health.

Regarding the first objective (to assess the levels of EE and their relationship with excessive internet usage and alcohol consumption): the average level of EE in the sample of students studied falls within the upper range of slightly emotional eaters (score between 6–10), very close to the range corresponding to emotional eaters (score between 11–20), according to the validation of the instrument conducted with a Spanish clinical population (participants in a weight-reduction program) (49). Although individuals with excess weight score higher on measures of EE than those within the “normal” weight category (59), our result is consistent with the estimated prevalence of EE found in earlier research, whereby 20–45% of adult non-clinical samples self-identify as emotional eaters (8, 60). According to accounts, these episodes frequently appear to be an attempt to prevent, manage, or deal with unpleasant feelings like sadness or anxiety (61). Approximately 44.7% of Spanish university students had mental distress suggestive of anxiety, while 13.5% showed indicators of depression, according to a survey involving approximately 706 individuals (62) and this would be in line with the prevalence data of EE found in this study. Additionally, it has been established that the EE propensity is linked to the tendency toward excessive internet use, but not to an increase in alcohol consumption. Consistently, it has been found that addictive internet use via smartphones in adolescents and young adults is associated with issues of control over eating, particularly with a greater tendency to eat automatically as a reaction to unpleasant feelings or emotions (25). Both excessive internet use and EE have been linked to symptoms of anxiety and depression in the general (non-clinical) population (7), and the likely connection between EE and excessive internet use is probably due to both being consequences of a lack of emotional regulation (27). There’s a proposal suggesting that alcohol consumption among university students could be influenced more by contextual factors, such as increased independence, reduced parental supervision, heightened social homogeneity, and the presence of alcohol-related social activities, rather than being primarily linked to psychological distress or difficulties in emotional regulation (63), and this would account for the absence of a correlation between alcohol consumption and excessive eating (EE) or internet overuse. On the other hand, if both EE and alcohol consumption can be understood as two different inadequate coping strategies for negative emotions, it may be that this stress response varies depending on variables such as gender (21), which, due to limitations related to sample size, we have not been able to consider in this pilot study.

Regarding objective 2 (verify the relationship between these behaviors, impulsivity and executive functioning assessed through a VR task). It is found that alcohol consumption is related to flexibility and working memory: lower cognitive flexibility and poorer performance in working memory during the VR task predict a higher tendency for alcohol intake. The relationship between alcohol consumption and executive issues in young population has been described in previous studies (20, 30), although other studies have not found this connection (64). These differences could suggest that self-report questionnaires or conventional neuropsychological tests do not accurately capture the complexity and dynamic character of real-life circumstances. To address these limitations, in our study is that the executive functioning of students has been assessed using an ecological VR task: the Ice Cream Test (ICT). Tools for neuropsychological assessments based on VR may provide improved validity and accuracy for evaluating a variety of cognitive skills, including executive functions (40, 58, 65, 66). There has not been any observed change in executive functions associated with the inclination toward EE or excessive internet use, in contrast to the effects seen with alcohol consumption. In line with the findings outlined in relation to objective 1, alcohol consumption seems to be linked to factors separate from those driving EE and internet overuse. However, both patterns of behavior, EE and excessive internet use, have been associated with increased impulsivity, which serves as a predictor in both instances. Previous studies have shown that decreased inhibitory control or impulsivity are linked to EE through a variety of different processes (67, 68). Impulsivity is defined as the tendency to act quickly in response to any stimulus (external or internal) without prior evaluation of all available information, hence, without considering the potential consequences of the action. From a neuropsychological perspective, impulsivity stems from a lack of inhibitory control, and response inhibition (which allows for the interruption or non-execution of inappropriate behavior) is a skill related to the integrity of the dorsolateral prefrontal cortex (69), an area still undergoing maturation in university students. Impulsivity plays a fundamental role in addictive behaviors, including non-substance addictions (44, 70). Impulsive behavior is maintained through positive reinforcement and is geared toward seeking pleasure and fulfilling individual needs. Impulsivity seems to underlie the onset of these behaviors, EE, and excessive/problematic internet use. In relation to eating behavior, there’s a proposed concept of an ‘eating continuum’ ranging from EE (considered non-pathological) to binge eating disorder (BED), suggesting an escalation in the severity of overeating behaviors coupled with a rise in deficits in emotion control and inhibition (47), and it would be important to take these factors, impulsivity and lack of inhibitory control, into account for preventive treatments for obesity or eating disorders.

Regarding objective three (to evaluate the role that depression and anxiety may have on these behaviors), trait depression and anxiety are related to excessive internet use and EE, but not to alcohol consumption. Depression emerges as a predictive variable for EE and internet addiction, but trait anxiety only predicts internet overuse, but not EE. A recent study finds that, of all the emotions described as triggers for episodes of loss of control over food intake (anxiety, depression, boredom, or happiness), depression was the factor most clearly associated with these episodes (3). Our results align with that conclusion, and from a clinical perspective, it appears that, in order to develop successful programs to prevent EE and obesity, sadness and depressive tendencies must be taken into consideration.

In our sample of university students, alcohol consumption is not associated with higher levels of anxiety or depression, reiterating that alcohol consumption among Spanish university students is driven by different factors compared to excessive eating (EE) and internet abuse. Emotional needs are not at all the sole motivator for university students to drink. Alcohol consumption is very common in this stage as most young people include alcohol in their leisure activities, and it forms a part of their recreational culture (71, 72).

Despite the phenomenological parallels between addictions and certain abusive acts (substance-free addictions by certain authors) (73, 74), our findings suggest that there are significant distinctions between alcohol intake and other substance-free addictive behaviors in terms of the components involved. Cognitive assessment via the VR task revealed neuropsychological impairment among individuals who consumed alcohol, but not among emotional eaters or those prone to excessive internet use. However, in the latter groups, other psychological factors such as impulsivity, anxiety, and depression appear to be significant contributors. Given the severe detrimental effects of internet overuse (behavior of growing prevalence) (75), both on personal and socio-familial aspects, it is important to consider these results for the implementation of prevention and intervention programs.

Further research is advised to better understand the various causal factors that lead to EE among college students. The study of the prevalence and frequency of EE during the formative years at university, as well as the exploration of the associated risk factors, is necessary. This knowledge will be necessary to support intervention techniques and programs run by public health and policy experts, and college students represent an important demographic for interventions aimed at preventing obesity. The use of VR as an assessment tool can evaluate participants’ performance in more ecological scenarios and provide more reliable results. But it is necessary to develop a more appropriate VR environment in the future for the assessment of cognitive functions, one that incorporates contexts relevant to participants’ concerns, such as EE, internet overuse, and alcohol consumption. This pilot study is the first step toward a more extensive study using this methodology.

This study has several limitations. Firstly, as we have already mentioned at the beginning of this introduction, the small sample size, largely due to the time and resources required to conduct the VR Tasks. A larger sample would allow us to include sociodemographic variables in the analysis, resulting in more broadly generalizable findings. Additionally, all participants were university students from Francisco de Vitoria University in Madrid. It would be desirable to compare the results obtained in this study with those obtained from students in other institutions and with non-student youth of the same age range. Moreover, longitudinal research is required. In reference to impulsivity, it would have also been desirable to assess it through a VR task rather than solely relying on a self-report questionnaire, although the Barratt Impulsiveness Scale shows good values of internal consistency in our study (*α* =0 0.70).

## Data availability statement

The raw data supporting the conclusions of this article will be made available by the authors, without undue reservation.

## Ethics statement

The studies involving humans were approved by Universidad Francisco de Vitoria. The studies were conducted in accordance with the local legislation and institutional requirements. The participants provided their written informed consent to participate in this study. Written informed consent was obtained from the individual(s) for the publication of any potentially identifiable images or data included in this article.

## Author contributions

CM-G: Writing – original draft, Writing – review & editing. MC-C: Writing – original draft, Writing – review & editing. EB-B: Conceptualization, Writing – original draft, Writing – review & editing.

## Footnotes

^1^ https://www.qualtrics.com
